# Effects of bone marrow-derived mesenchymal stem cells transplanted via the portal vein or tail vein on liver injury in rats with liver cirrhosis

**DOI:** 10.3892/etm.2015.2232

**Published:** 2015-01-29

**Authors:** YING-MING SONG, CHANG-HONG LIAN, CHENG-SONG WU, AI-FANG JI, JUAN-JUAN XIANG, XIAO-YAN WANG

**Affiliations:** 1 Department of General Surgery, Heping Hospital, Changzhi Medical College, Changzhi, Shanxi 046000, P.R. China; 2 Department of Gastroenterology, The Third Xiangya Hospital, Central South University, Changsha, Hunan 410013, P.R. China; 3 Cancer Research Institute, Central South University, Changsha, Hunan 410078, P.R. China

**Keywords:** rats, bone marrow mesenchymal stem cells, liver injury, transplantation route

## Abstract

The aim of the present study was to compare the effects of bone marrow-derived mesenchymal stem cells (BMSCs) transplanted via the portal vein or tail vein on liver injury in rats with liver cirrhosis. BMSCs were isolated from rat bone marrow and labeled with green fluorescent protein (GFP). Then, the labeled BMSCs were injected into rats with liver injury via the portal vein or tail vein. Two weeks after transplantation, three rats in each group were sacrificed to test the distribution of GFP in the liver and the serum levels of alanine aminotransferase (ALT), aspartate aminotransferase (AST) and albumin. Six weeks later, the remaining rats were sacrificed, and serum ALT, AST, albumin, hyaluronic acid (HA), laminin (LN) and procollagen type III (PC-III) levels were measured. The expression of albumin in the liver was analyzed by immunohistochemistry. Two weeks after BMSC transplantation, GFP-positive cells were detected in the livers of rats with BMSCs transplanted via the portal vein and tail vein. Compared with pre-transplantation levels, the ALT levels of the groups with BMSC transplantation via the portal vein and tail vein were significantly decreased after two and six weeks of BMSC transplantation (P<0.05), whereas the AST and albumin levels were not significantly different at two weeks after BMSC transplantation in the two groups (all P>0.05). However, the AST and albumin levels were significantly reduced at six weeks after BMSC transplantation (all P<0.05). At six weeks after BMSC transplantation, the serum HA, LN and PC-III levels in rats transplanted with BMSCs via the portal vein or tail vein had decreased significantly (all P<0.05), as compared with the levels prior to BMSC transplantation. BMSCs transplanted via the portal vein and tail vein achieved similar improvements in liver function in rats with liver cirrhosis, which suggests that peripheral venous administration is a convenient and effective route for BMSC transplantation.

## Introduction

Liver injury due to chemical damage or viral infection often leads to liver fibrosis and liver failure, and this may lead to an impairment of liver function. Liver transplantation is the most effective treatment for liver cirrhosis, with the ability to improve the quality of life and prognosis of patients with liver cirrhosis. However, extensive clinical application of the technique is limited by the lack of donor organ availability (1). Thus, the investigation of other treatments and therapies for cirrhosis is necessary. Bone marrow mesenchymal stem cells (BMSCs) can differentiate into hepatocyte-like cells, and support organ regeneration processes. Moreover, BMSCs are safer than embryonic stem cells to use *in vivo* due to their higher chromosomal stability and lower tendency to form neoplasms in the recipient host. Therefore, BMSC transplantation has become a novel therapeutic strategy for liver injury (2–4). Certain clinical studies have suggested that BMSC transplantation is an effective treatment for patients with severe liver disease (5–7). As a new treatment, certain issues remain to be resolved in the application of BMSC transplantation in the treatment of liver cirrhosis, such as the efficiencies of various transplantation paths, the optimum cell counts of BMSC transplantation and the timing of transplantation. In a previous study, Sun *et al* (4) compared the efficiencies of BMSC transplantation by the portal vein, abdominal cavity and liver, and the results indicated that the best efficiency of BMSC transplantation was obtained via the portal vein. Currently, BSMCs are generally transplanted via the portal vein in patients with liver diseases as the first-pass effect in the liver is much higher than that when the transplantation is via other routes (8–10). However, BSMC transplantation via the portal vein could transiently increase venous pressure and create a venous embolism, which would aggravate liver injury (11). Moreover, puncture of the portal vein is a relatively difficult procedure and not convenient to conduct in the clinic. Therefore, it is important to identify effective, safe and convenient administration methods for BMSC transplantation.

One convenient mode of administration of BMSC transplantation is via a peripheral vein; however, the therapeutic effect of BMSC transplantation via a peripheral vein is not clear. Several studies have shown that the colonization and differentiation of BMSCs in the organ are mainly induced by the microenvironment in the injured organ, indicating that a peripheral vein may be an alternative administration site for BMSC transplantation (12–14). However, it is unclear whether BMSCs transplanted via a peripheral vein colonize in the liver and exert their functions properly. In this study, BMSCs were transplanted via the portal vein and via a peripheral vein in rats with liver cirrhosis, and the therapeutic effect of BMSC transplantation on liver injury and liver fibrosis in the rats was studied. The results were analyzed to determine whether BMSC transplantation via a peripheral vein is an effective and convenient BMSC administration route for liver cirrhosis.

## Materials and methods

### Materials

#### Animals

A total of 58 male Sprague-Dawley (SD) rats, 6–8 weeks of age, weighing 130–150 g were purchased from the Zoology Section of Changzhi Medical College (Changzhi, China). The rats were bred under specific pathogen-free conditions in the Zoology Section of Changzhi Medical College.

#### Cells

293T cells were purchased from Aiyan Biological Technology Co., Ltd. (Shanghai, China). The cells were cultured in high-glucose Dulbecco’s modified Eagle’s medium (DMEM) supplemented with 10% fetal bovine serum (FBS). Cells were grown at 37°C under 5% CO_2_.

#### Main reagents and antibodies

DMEM, FBS and 0.25% trypsin were purchased from Hyclone (Shanghai, China). Assay kits to measure alanine aminotransferase (ALT), aspartate aminotransferase (AST) and albumin were purchased from Zhangjiang Biotechnology Co., Ltd. (Shanghai, China). Percoll was purchased from Amersham Pharmacia Biotech (Piscataway, NJ, USA). LentitopoLuc-green fluorescent protein (GFP), was obtained from the Cancer Research Institute of Central South University (Changsha, China) (11). The ViraPower plasmid system and liposomes were purchased from Invitrogen Life Technologies (Carlsbad, CA, USA). Hematoxylin and eosin, and Masson stains were purchased from Yansheng Biochemical Reagents Co., Ltd. (Shanghai, China). Polyclonal goat anti-mouse albumin antibodies were purchased from Abcam (Cambridge, UK). Radioimmunoassay kits used to determine the levels of the liver fibrosis markers hyaluronic acid (HA), laminin (LN) and procollagen type III (PC-III) were purchased from Haiyan Medical Biotechnology Co., Ltd. (Shanghai, China).

### Methods

#### Isolation and culture of rat BMSCs

Rats were anesthetized by an intraperitoneal injection of 10% hydral. The femurs and tibias were removed, and the two terminal regions of these bones were cut and flushed with DMEM to collect cells. The cell mixture was centrifuged at 150 × g for 10 min, to prepare a single-cell suspension, and the supernatant was discarded. The BMSCs were isolated with Percoll and cultured in DMEM supplemented with FBS for 48 h. After removing the cell suspension, adherent BMSCs were obtained. The BMSCs were grown to passage 3 and were used in experiments once they reached 80% confluence.

#### BMSC labeling

For labeling, 10 μg DNA of proteolipid protein 1 (RC225379; Amsbio, Abingdon, UK), 15 μg DNA of proteolipid protein 2 (MG224890; Amsbio), 7.5 μg DNA of vesicular stomatitis Indiana virus G protein (HT-pack; Amsbio) and 20 μg DNA of LentitopoLuc-GFP were added to serum-free DMEM. Then, 30 μl liposomes were added, and the mixture was placed on ice for 20 min. The mixture of DNA and liposomes was added to 293T cells (5×10^6^). After 12 h, the medium was replaced with DMEM and the cells were cultured for a further 72 h. The supernatant was collected to determine the virus titer, and then the supernatant was filtered through a 0.45-μm filter and added to BMSCs, which were cultured for 48 h. The transfection efficiency of GFP was observed under a fluorescent microscope (FSX100; Olympus Corporation, Tokyo, Japan) (5).

#### Establishment of liver cirrhosis in rats

To establish the liver cirrhosis rat model, 50:50 (v/v) CCl_4_ and vegetable oil mixture was injected subcutaneously into the 58 adult male SD rats twice a week for 10 weeks. Three rats from each group were randomly selected and the liver was stained with hematoxylin/eosin (H&E) and Masson stains to confirm the establishment of liver cirrhosis.

#### H&E and Masson staining

In the H&E staining process, paraffin-embedded liver sections (4 μm) were deparaffinized with xylene, dehydrated in alcohol and washed. The sections were stained in hematoxylin solution for 5 min and then washed, followed by differentiation in 1% acid alcohol for 30 sec, and washing for 10 min. The sections were then counterstained with eosin-phloxine solution for 3 min, washed and dehydrated. Finally, the slides were cleared in xylene and mounted with a xylene-based mounting medium.

For Masson staining, the slides were deparaffinized, dehydrated, and then washed and stained in Weigert’s iron hematoxylin for 5 min. Next, the slides were washed, stained in Biebrich scarlet acid fuchsin solution for 5 min, washed and differentiated in 1% phosphomolybdic-phosphotungstic acid solution for 5 min. The sections were transferred to aniline blue solution and stained for 5 min, and then differentiated in 1% acetic acid solution for 1 min. Finally, the sections were washed, dehydrated, cleared in xylene and mounted with resin mounting medium.

#### Treatments

Overall, 48 rats with cirrhosis (7 rats died during model establishment) were divided into four equal groups of 12 rats per group to receive injection of BMSCs or PBS via the portal vein and tail vein, respectively.

#### Transplantation of BMSCs

In each group, the BMSCs were resuspended in 0.1 mol/l phosphate-buffered saline (PBS) at a concentration of 10^7^ cells/ml, and each rat was injected with 500 μl GFP-labeled BMSCs or the same volume of PBS. For the portal vein groups, rats were anesthetized with 10% chloral hydrate and the abdominal cavity was opened to expose the portal vein, into which BMSCs or PBS were injected. After removing the needle, pressure hemostasis was applied for 1 min.

#### Blood and tissue collection and processing

Tail vein blood samples were collected shortly prior to transplantation and used to measure serum ALT, AST, albumin, HA, LN and PC-III levels. Two weeks after transplantation, three rats in each group were sacrificed, and the liver was removed, fixed and sectioned. Liver samples were observed under a fluorescence microscope (Olympus FSX100) to determine the distribution of GFP in the liver. At the same time, blood samples were taken to measure serum ALT, AST and albumin levels. At six weeks after transplantation, all the remaining rats in each group were sacrificed. Cardiac puncture blood samples were obtained to measure ALT, AST, albumin, HA, LN and PC-III levels. The liver was fixed, sectioned and albumin expression was determined by immunohistochemistry.

#### Immunohistochemistry

The rat liver tissue sections were deparaffinized, dehydrated, washed and subjected to citrate antigen retrieval. After incubation in hydrogen peroxide, the sections were blocked in serum-free blocking medium, and then incubated with anti-albumin antibody (1:1,000 dilution) overnight at 37°C. The sections were then washed and incubated with 200 μl rabbit anti-goat immunoglobulin G (H&L) horseradish peroxidase-conjugated secondary antibody (1:400 dilution; cat. no. ab6741, Abcam) for 2 h at room temperature. Following this, the sections were washed and incubated with avidin-biotin complex [UltraSensitive™ SAP (mouse/rabbit) IHC kit (Fuzhou Maixin Biotechnology Development Co., Ltd., Fujian, China)] at room temperature, washed and stained with 3,3′-diaminobenzidine (DAB) Detection (Streptavidin/Biotin) kit (Fuzhou Maixin Biotechnology Development Co., Ltd.). Finally, the slides were dehydrated, cleared with xylene and mounted with permanent mounting medium.

The relative expression of albumin was determined in terms of the degree of staining and the number of positive cells. The degree of staining was classified as follows: 0, no staining; 1, light orange-stained cells; 2, yellow-stained cells; and 3, brown-stained cells. The number of stained cells was quantified as follows: 0, <5% positive cells; 1, 5–25% positive cells; 2, 26–50% positive cells; 3, 51–75% positive cells; and 4, >75% positive cells. The sections were graded by adding together the scores for degree and extent of staining, and the immunohistochemical data were classified as follows: 0, negative staining (−); 1 or 2, weakly positive (+); 3–5, moderately positive (++); 6 or 7, strongly positive (+++).

### Data analysis

The statistical software SPSS version 16.0 (SPSS, Inc., Chicago, IL, USA) was applied to analyze the data. Data of ALT, AST, albumin, HA, LN and PC-III are shown as the means ± standard deviations. Groups were compared using repeated measures analysis of variance, and the albumin immunohistochemistry data were analyzed by nonparametric test. Values of P<0.05 are considered statistically significant.

## Results

### Efficiency of GFP transfection in 293T cells

**Figure 1:**
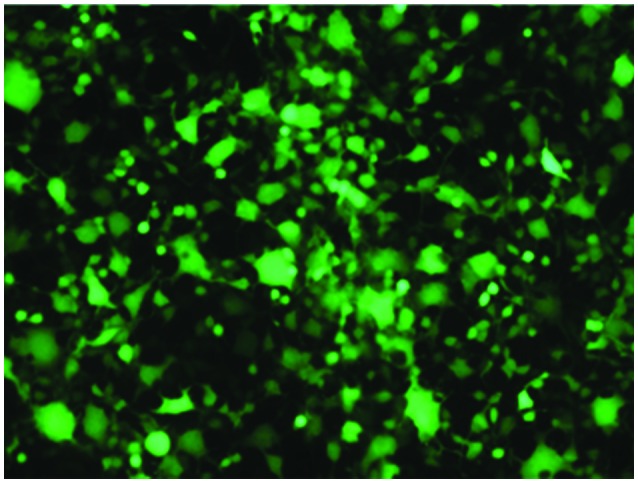
Confirmation of green fluorescent protein expression in 293T cells (original magnification, ×100).

The 293T cells were co-transfected with three plasmids and GFP. At 48 h after transfection, the efficiency of the transfection was observed under a fluorescence microscope (Fig. 1). All the cells examined expressed GFP, and the virus titer was 2.23×10^9^.

### Labeling of BMSCs

**Figure 2:**
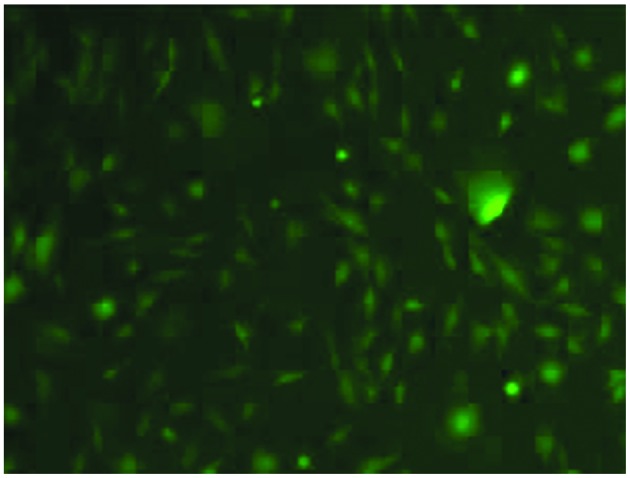
Observation of green fluorescent protein-labeled bone marrow mesenchymal stem cells under a fluorescence microscope (original magnification, ×100).

BMSCs were cultured with medium collected from 293T cells at 48 and 72 h after transfection with GFP. After 48 h of culture, GFP-positive BMSCs were observed by fluorescence microscopy, confirming that the BMSCs were labeled with GFP (Fig. 2).

### Characteristics of rats with liver cirrhosis

**Figure 3:**
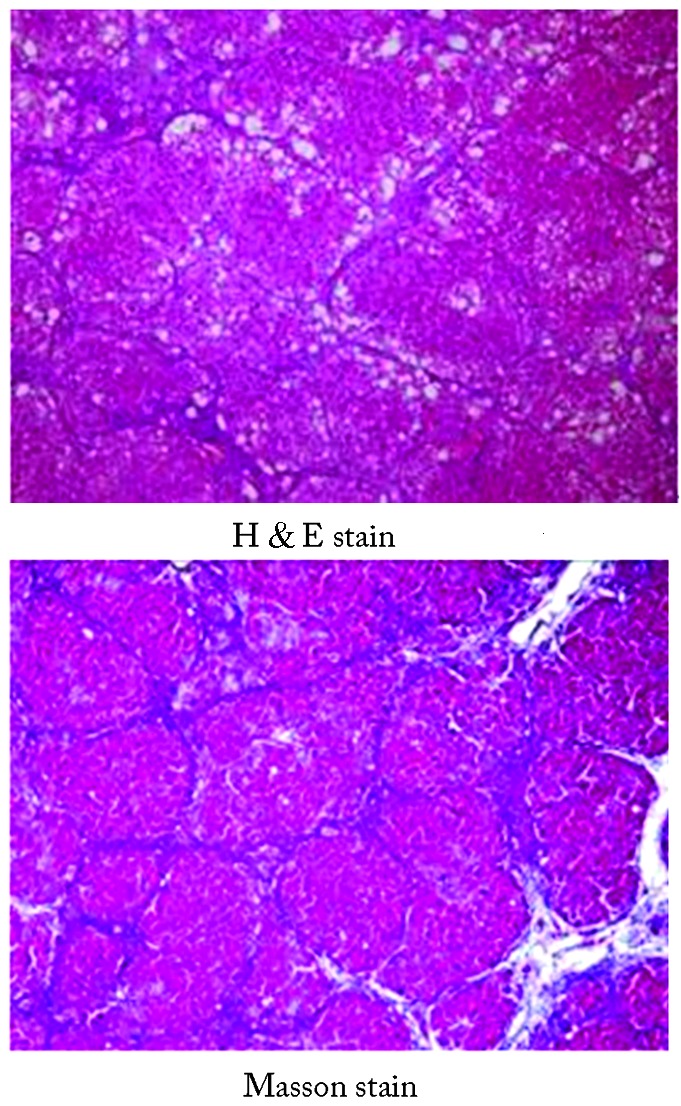
Hematoxylin and eosin (H&E) and Masson staining of the liver tissue of rats with liver cirrhosis (original magnification, ×40).

Following the subcutaneous injection of 50:50 (v/v) CCl_4_/vegetable oil mixture into the rats, they developed anorexia and were emaciated. Their urine was dark yellow in color. After 10 weeks of CCl_4_ administration, seven rats died. Using a random-digits table, three rats were randomly selected for liver staining with H&E and Masson stains. In these rats, the formation of hepatic fibrosis and pseudolobules, enlargement of the hepatocytes, and focal necrosis were observed, which confirmed the successful establishment of a rat model of liver cirrhosis (Fig. 3).

### Hepatic distribution of BMSCs in rats with liver cirrhosis

**Figure 4:**
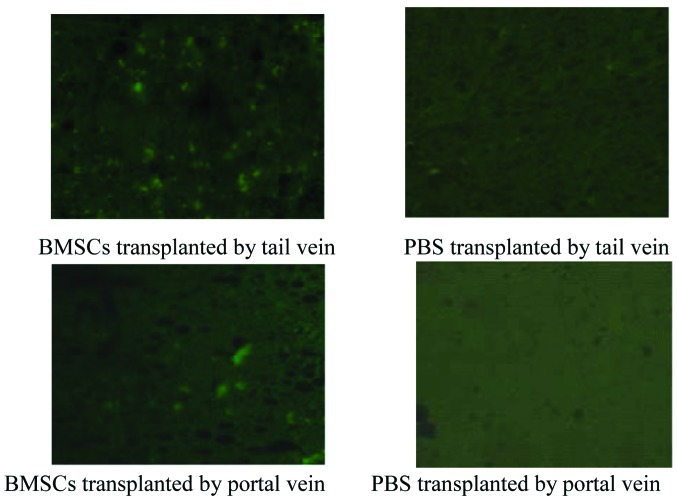
Distribution of BMSCs transplanted via the tail vein and portal vein in the pathologically changed liver. BMSC, bone marrow mesenchymal stem cell; PBS, phosphate-buffered saline.

Two weeks after transplantation, three rats from each group were sacrificed, and the hepatic distribution of BMSCs was determined. The expression of GFP was visible in the livers of the rats transplanted with BMSCs via the portal vein or via tail vein; however, no GFP expression was observed in the livers of the rats injected with PBS (Fig. 4). These results indicate that BMSCs transplanted via the portal vein or tail vein are able to colonize the liver.

### Effects of BMSC transplantation on markers of liver injury

The changes in ALT, AST and albumin levels were measured prior to and at two and six weeks after BMSC transplantation via the portal vein or tail vein. Serum ALT levels decreased significantly at two and six weeks after BMSC transplantation via both routes (P<0.05; Table I). AST and albumin levels measured prior to and at two weeks after BMSC transplantation were not significantly different in each group (P>0.05; Tables II and III). However, the serum AST level was significantly lower and the serum albumin level was significantly higher at six weeks after transplantation compared with the levels measured prior to BMSC transplantation (both P<0.05).

Serum ALT levels in rats at two and six weeks after BMSC transplantation via the portal vein or via tail vein were significantly lower than those in rats injected with PBS via the portal vein or via tail vein (P<0.05). Furthermore, the serum level of AST was significantly lower and the serum level of albumin was significantly higher at six weeks in the rats that had undergone BMSC transplantation compared with rats injected with PBS (P<0.05). There were no significant differences in serum ALT, AST or albumin levels between rats transplanted with BMSCs via the portal vein or via tail vein either prior to or following BMSC transplantation (all P>0.05).

The serum HA, LN and PC-III levels in the rats six weeks after BMSC transplantation via the portal vein or tail vein were significantly decreased compared with those measured prior to BMSC transplantation (all P<0.05; Table IV). The levels in BMSC transplanted rats were also significantly lower than those in rats injected with PBS. However, there were no significant differences between rats transplanted with BMSCs via the portal vein or tail vein (all P>0.05). There were also no differences in the levels of HA, LN or PC-III at six weeks after the injection of PBS compared with those measured prior to injection.

### Effects of BMSC transplantation on hepatic albumin expression

**Figure 5:**
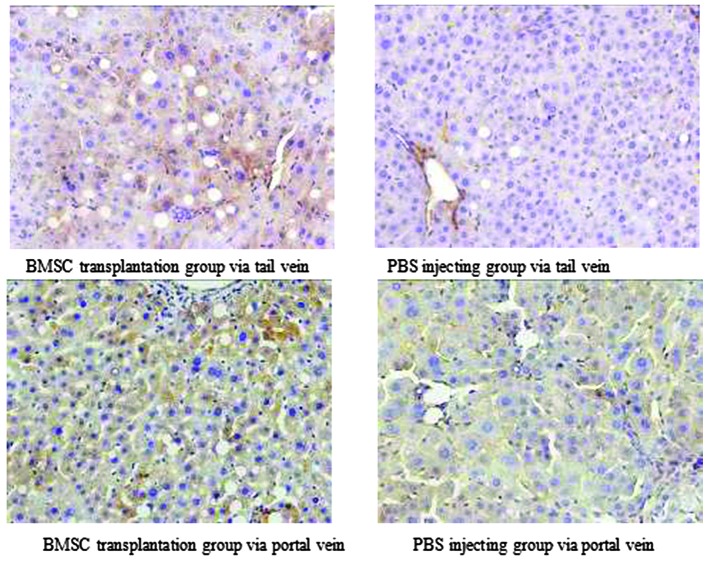
Effects of BMSC transplantation on albumin expression in rat liver tissue (original magnification, ×200). BMSC, bone marrow mesenchymal stem cell; PBS, phosphate-buffered saline.

Immunohistochemistry was used to assess hepatic albumin expression in the four experimental groups. Albumin protein expression in the cytoplasm was represented by the accumulation of orange or brown particles (Fig. 5). Nonparametric statistical tests revealed a significant difference in hepatic albumin expression between the two BMSC transplanted groups compared with their matched control groups (P<0.05). However, there was no difference in albumin expression between rats transplanted with BMSCs via the portal vein or tail vein (P>0.05; Table V).

## Discussion

Liver cirrhosis is a common disease in China. Patients with aggravation of liver injury often show serious complications, such as liver failure and hepatic encephalopathy (15). The mortality rate associated with this disease is extremely high and there are no effective treatment methods for liver cirrhosis, with the exception of liver transplantation, which is limited by the availability of donor livers. BMSCs have the capacity of self-renewal and can differentiate into multiple cell types. *In vitro* studies have shown that BMSCs can be induced to differentiate into hepatocyte-like cells following exposure to hepatocyte growth factor, fibroblast growth factor-4 and epidermal growth factor (16–20), offering a novel strategy for the treatment for terminal liver disease. Therefore, BMSC transplantation is increasingly being used in the treatment of patients with severe liver disease, and much progress has been made in the development of this therapeutic approach (21). BMSC transplantation via the portal vein is likely to become a universal method due to the first-pass effect of the portal circulation. However, puncture of the portal vein is relatively difficult and may increase the risk of liver injury. Our previous, unpublished research revealed that the colonization of BMSCs in the liver is strongly associated with chemotactic processes underlying organ injury. Therefore, the identification of alternative transplantation routes is essential to avoid these issues. Consequently, the present study sought to investigate whether BMSCs transplanted via a peripheral vein were capable of colonizing in the liver and were functional.

In this study, GFP-labeled BMSCs were transplanted or PBS injected via the portal vein or tail vein into rats with experimental liver cirrhosis. The colonization of BMSCs in the liver and the effects of BMSC transplantation on markers of hepatic injury and fibrosis were examined, and these parameters were compared between the two routes of transplantation. Extensive green fluorescence corresponding to GFP-labeled BMSCs was observed in the livers of rats at two weeks after BMSC transplantation via the portal vein and via the tail vein, indicating that the peripherally transplanted BMSCs were able to colonize the injured liver, in a similar manner to that achieved by portal vein injection. This colonization may be at least partly associated with chemotactic events in liver injury (22). The effects of both routes of BMSC transplantation on markers of liver injury (i.e., ALT, AST, albumin, HA, LN and PC-III) were then compared. Serum ALT levels improved significantly at two and six weeks after BMSC transplantation in both groups as compared with levels measured prior to transplantation. Serum AST and albumin levels measured at two weeks after BMSC transplantation were not significantly different compared with the levels measured before transplantation. However, at six weeks after transplantation, serum AST and albumin levels had improved significantly in both groups; they decreased and increased, respectively. Serum ALT, AST and albumin levels were significantly better at six weeks after BMSC transplantation compared with those in the PBS-injected control groups. Serum HA, LN and PC-III levels were significantly lower at six weeks after BMSC transplantation via the portal vein or tail vein compared with levels measured prior to BMSC transplantation or in the control groups. Immunohistochemical analysis of liver sections showed that hepatic albumin expression was markedly enhanced at six weeks after BMSC transplantation, consistent with increases in serum albumin levels, indicating that BMSCs transplanted via the portal vein or tail vein can improve liver function and reduce liver fibrosis to a similar extent in rats with liver cirrhosis. Notably, there were no differences in serum ALT, AST, albumin, HA, LN or PC-III levels between the two groups of rats. These effects on liver function may be achieved by the transplanted BMSCs colonizing in the liver and then differentiating into hepatocyte-like cells in response to the liver’s microenvironment. The hepatocyte-like cells may suppress inflammatory activity by secreting growth factors and cytokines, and decrease hepatocyte apoptosis (23–27).

These results indicate that BMSCs transplanted via the portal vein or tail vein can colonize in the liver to improve liver function and reduce liver fibrosis. Notably, there were no differences in these effects of BMSCs transplantation between the two administration methods. Therefore, BMSC transplantation via a peripheral vein is indicated to be an effective, safe and practical method for treating liver injury.

## Figures and Tables

**Table I:** Effects of BMSC transplantation on serum ALT levels (mean ± standard deviation).

	Serum ALT at different time points (U/l)		
	
Groups	Before transplantation	Two weeks after transplantation	Six weeks after transplantation
BMSC transplantation via tail vein	148.3±15.6	127.3±13.6^a^,^b^	84.7±9.9^a^,^b^
PBS injection via tail vein	142.4±14.4	139.3±15.3	128.1±13.4
BMSC transplantation via portal vein	157.6±18.2	130.5±14.8^a^,^b^	92.4±11.7^a^,^b^
PBS injection via portal vein	160.7±14.6	155.1±12.8	143.2±15.2

BMSC, bone marrow mesenchymal stem cell; ALT, alanine aminotransferase; PBS, phosphate-buffered saline.

a P<0.05 vs. before transplantation.

b P<0.05 vs. the respective control (PBS) group.

**Table II:** Effects of BMSC transplantation on serum AST levels (mean ± standard deviation).

	Serum AST at different time points (U/l)		
	
Groups	Before transplantation	Two weeks after transplantation	Six weeks after transplantation
BMSC transplantation via tail vein	224.5±20.1	201.6±19.3	165.4±16.5^a^,^b^
PBS injection via tail vein	218.7±22.6	209.8±21.8	202.4±19.7
BMSC transplantation via portal vein	228.4±25.2	206.3±21.5	170.6±20.5^a^,^b^
PBS injection via portal vein	222.3±22.3	215.2±21.8	211.3±20.6

BMSC, bone marrow mesenchymal stem cell; AST, aspartate aminotransferase; PBS, phosphate-buffered saline.

a P<0.05 vs. before transplantation.

b P<0.05 vs. the respective control (PBS) group.

**Table III:** Effects of BMSC transplantation on serum albumin levels (mean ± standard deviation).

	Serum albumin at different time points (g/l)		
	
Groups	Before transplantation	Two weeks after transplantation	Six weeks after transplantation
BMSC transplantation via tail vein	27.2±3.4	28.4±2.9	32.5±2.5^a^,^b^
PBS injection via tail vein	26.3±3.0	26.9±2.2	27.5±2.4
BMSC transplantation via portal vein	26.2±2.6	27.7±2.5	31.9±2.0^a^,^b^
PBS injection via portal vein	28.1±3.5	28.8±2.7	29.1±2.6

BMSC, bone marrow mesenchymal stem cell; PBS, phosphate-buffered saline.

a P<0.05 vs. before treatment.

b P<0.05 vs. the respective control (PBS) group.

**Table IV:** Effects of BMSC transplantation on serum HA, LN and PC-III levels of rats (mean ± standard deviation).

	HA (ng/ml)		LN (ng/ml)		PC-III (ng/ml)	
			
Groups	Before transplantation	Six weeks after transplantation	Before transplantation	Six weeks after transplantation	Before transplantation	Six weeks after transplantation
BMSC transplantation via tail vein	283.49±15.41	198.44±14.92^a^,^b^	97.45±7.89	62.41±6.98^a^,^b^	197.42±16.09	137.43±12.69^a^,^b^
PBS injection via tail vein	272.33±13.94	258.94±12.23	83.90±8.29	73.19±5.63	206.91±14.22	192.95±12.82
BMSC transplantation via portal vein	265.27±12.15	188.73±13.05^a^,^b^	101.57±10.56	70.57±6.59^a^,^b^	189.79±12.53	129.17±10.54^a^,^b^
PBS injection via portal vein	280.61±14.35	264.48±12.27	89.58±9.73	76.38±8.73	190.48±13.76	184.18±13.79

BMSC, bone marrow mesenchymal stem cell; HA, hyaluronic acid; LN, laminin; PC-III, procollagen type III.

a P<0.05 vs. the value before treatment in the same group.

b P<0.05 vs. the value in the respective control (PBS) group.

**Table V:** Effects of BMSC transplantation on albumin expression in rat liver tissue.

Group	−	+	++	+++
BMSC transplantation via tail vein^a^	0	1	4	6
PBS injection via tail vein^b^	0	4	5	1
BMSC transplantation via portal vein^a^	0	2	3	5
PBS injection via portal vein^d^	0	3	7	0

BMSC, bone marrow mesenchymal stem cell; PBS, phosphate-buffered saline.

a P<0.05 vs. the respective control (PBS) group. There was no significant difference between the two BMSC transplantation groups.
